# Designing with Protocells: Applications of a Novel Technical Platform

**DOI:** 10.3390/life4030457

**Published:** 2014-09-05

**Authors:** Rachel Armstrong

**Affiliations:** Architecture, Planning and Landscape, University of Newcastle, Newcastle-upon-Tyne, NE1 7RU, UK; E-Mail: grayanat@yahoo.co.nz; Tel.: +44-795-464-0759

**Keywords:** Bütschli droplets, architecture, design, technical system, assemblage, process philosophy, living buildings, ecological

## Abstract

The paper offers a design perspective on protocell applications and presents original research that characterizes the life-like qualities of the Bütschli dynamic droplet system, as a particular “species” of protocell. Specific focus is given to the possibility of protocell species becoming a technical platform for designing and engineering life-like solutions to address design challenges. An alternative framing of the protocell, based on process philosophy, sheds light on its capabilities as a technology that can deal with probability and whose ontology is consistent with complexity, nonlinear dynamics and the flow of energy and matter. However, the proposed technical systems do not yet formally exist as products or mature technologies. Their potential applications are therefore experimentally examined within a design context as architectural “projects”—an established way of considering proposals that have not yet been realized, like an extended hypothesis. Exemplary design-led projects are introduced, such as The Hylozoic Ground and Future Venice, which aim to “discover”, rather than “solve”, challenges to examine a set of possibilities that have not yet been resolved. The value of such exploration in design practice is in opening up a set of potential directions for further assessment before complex challenges are procedurally implemented.

## 1. Introduction

With the rise of the biotechnological age, forays have been made into working with lively matter as a technical system, which are informed by the study of process philosophy [1]. This platform can perform equivalent kinds of work that we would usually associate with the performance of machines, such as producing heat, or making a structure. While such processes may be carried out by our native biological systems—or “*nanotechnology that works*” [2]—in the last few decades, a range of dynamic chemical and molecular systems that are capable of producing autonomous activities, have sprung to the fore, with potential application in generating novel design and engineering solutions, such as self-healing materials [3]. A growing range of smart materials available to designers called protocells—which may be thought of as a particular kind of life-like smart chemistry—raise far-reaching questions about the potential applications of this new technical system. This does not merely refer to the instrumental value of the platform, in other words, the kinds of tasks that can be performed by utilizing the properties of life-like agents but also to the ontological implications of this technology. Life-like systems may be regarded differently than machines, with different outcomes and impacts and constitute a technical platform through which many ideas and observations about their potential converge. Indeed, the range of entities that may currently be recognized as protocells embody a spectrum of design strategies—from Freeman Dyson’s dustbin bag metabolism [4] to Pier Luigi Luisi’s dynamic oil droplets [5] and Jack Szostak’s RNA-containing vesicles [6]. Each of these different species of protocell agents embody a unique set of principles that not only reposition humanity’s relationship with “life” but also offer new approaches for design practice. This paper addresses a range of approaches through which protocells may achieve their design and engineering goals by considering their ontology as a technical system and what this means when applying these agents in practice.

### 1.1. Protocells as a Unique Technical System

Our industrial age is founded on Enlightenment principles that exemplify the notion that matter is a passive substance upon which empowered agencies may act, such as humans, or forms of machine intelligence. While this is an effective approach in mass manufacturing products, industrialization does not recognize the dynamic complexity of the natural world and in fact, its processes are highly damaging in this respect. Over the course of the 20th century the practice of chemistry in particular has become increasingly equated with industrial manufacturing systems, which rid matter of its innate agency by abstracting and de-complexifing matter by for example, transforming mineral ores into metals, or devitalizing living things, like trees, into inert materials, such as planks of wood. Indeed, industrial practices have been condemned by political commentators, such as Michel Serres, as a “*revolution operating on matter*” [7] and by environmentalists, such as Rachel Carson, as being responsible for widespread ecological destruction [8]. A different way of framing the material and technical potential of lively chemistry is needed for 21st century techniques, so that the full spectrum of chemical phenomenon that includes, for example, transitional and intermediary states, may be theoretically and practically addressed.

Gordon Pask and Stafford Beer experimented with some of the design possibilities offered by empowered materials, by considering them as cybernetic systems. Pask explored crystal formation in response to street sounds [9] (pp. 185–212) and Beer used the microorganism daphnia—a microscopic crustacean commonly known as the “water flea”—as well as entire pond ecologies as alternative media for cybernetic systems [10]. However, their ability to manipulate material systems through positive and negative feedback loops alone, became quite limited from a design and engineering perspective, particularly since the emerging field of biotechnology was not mature. However, over the last few decades the chemical operations of lively materials have been characterized in fine detail to the point where biochemical processes within living systems—called bioprocesses—are not just a material substrate, but, also, simultaneously double up as the technical system through which these substances are instrumented.

Protocells represent a turning point in the evolution of life-like technologies, because they already demonstrate a remarkable range of life-like properties, which have been constructed from a bottom up perspective from fundamental ingredients. In some cases, such as the Bütschli droplet system, they are able to move around their environment, sense it, operate as populations of agents, and even produce microstructures [11].

Moreover, the protocell platform strives to become indistinguishable from life itself and represents a unique convergence between what Kevin Kelly calls “*the born and the made”* [12]. Such technical convergences are of interest to national funding bodies to produce modes of production with far-reaching effects for society and the economy. The convergence of the emerging technologies nanotechnology, cognitive technologies, biotechnology, and information technology—have been of particular interest as they represent a material singularity, which constitutes the so-called NBIC convergence [13], where tipping points in the ordering of matter and information are reached. To date, the outcomes of such convergences have failed to deliver radical new impacts since they manifest as technical collages framed by Enlightenment principles that are exemplified by projects, such as “cyberplasm”, which is a hybrid system formed by discreet components derived from synthetic biology and information technologies [14]. In this sense, protocells differ to such technological amalgamations as they represent an integrated platform, whereby the software and hardware of the technology do not need to be bolted together but are already multiscalar assemblages that integrate information and materiality, and, as such, have the potential to provide a connecting matrix in which other technical systems may also be seamlessly integrated.

However, rather than addressing the technical aspects of protocell “design” through the actual composition and construction of the platforms through which “life” itself may eventually be manifest, this paper offers a designer’s perspective on the applications of protocells. In other words, its focus is not on the incremental technical and scientific advances needed to advance the performance of protocells towards a state that we may ultimately recognize as being indistinguishable from “life”. Rather, it considers how we may work with the existing properties of the range of chemistries that are currently identified as protocells, which are considered as part of a broader technical system, which include agents from simple dynamic droplets [15] to complex, fully artificial cells that meet the Chemoton model criteria—of container, metabolism, and information [16]—that do not yet exist [17]. These various integrated chemical operating systems are differentially able to offer a range of material transformations that may perform useful work. In other words, it addresses the title of this special issue by considering the value of designing with life-like strategies and the methods through which they may be instructed, rather than focusing on how life itself may be synthesized.

The starting point of my essay therefore takes a design perspective to examine the uses of life-like chemistries in culturally meaningful contexts, where the general public can view their relevance and reflect on their potential.

### 1.2. What Are Protocells?

Protocells are “primordial molecular globules, situated in the environment through the laws of physics and connected through the language of chemistry” [18].

Protocells have not been reported as being found spontaneously in Nature and are therefore entirely artificial, being the product of human observation, design and engineering goals. Protocell species are prototypes of primitive cells, whose primordial nature is related to the bottom-up approach taken towards development of an artificially constructed cell [19]. Owing to a growing interest in the field, a range of approaches, definitions and types of protocell species exist. The controversial and ambiguous nature of the term invites a broad range of definitions. Its etymology implies a chemical agent that possesses some of the formal qualities of “life”, such as movement, sensitivity, metabolism, growth, or repair, yet is not given the full status of being fully alive [20]. However, sometimes, the term has been used interchangeably with “vesicle” [6], while at other times it may indicate fully artificial chemical cells capable of replication [17,19]. In this current investigation, the term is used to refer to real cell models that to date, exhibit a range of recognizably life-like qualities but do not qualify as being fully “alive”. Specifically, the Bütschli system—a water in oil dynamic droplet system, is used as a model to reflect on the potential of a broader portfolio of agents that may also be described as “protocells”.

### 1.3. What Are the Properties of Protocells that Are of Interest to Design?

Protocells represent and embody the convergence of natural and artificial systems. They are characterized by their striking life-like qualities, which potentially have great value in design as they represent a platform that is simultaneously “natural” in terms of its emergent spontaneity and also their artificiality, since they are also partly designed and deliberately constructed. Such contradictory qualities may be meaningfully employed as agents of design when their properties and interactions can be choreographed through deliberate interventions. This may be achieved by applying natural computing techniques.

The term natural computing was inspired by Alan Turing’s interest in the computational powers of Nature [21,22]. It refers to a very broad set of practices and is relatively recently established, so its application has been developed and interpreted according to the aims of the various participating research groups. Researchers include Martin Hanczyc, at the University of Trento [15], Lee Cronin, at the University of Glasgow [23], Klaus-Peter Zauner, at the University of Southampton [24], Gabriel Villar, at the University of Oxford [25], and Andy Adamatzky, at the University of the West of England [26]. The main goal of natural computing is to develop programmable, life-like systems using a spectrum of platforms to better understand and reflect the properties of living things, such as adaptation, learning, evolution and growth. Research practices include the study of biomimicry in digital computing that notionally engages with material processes, mainly through representations in “genetic algorithms”. However, the concerns of the investigation presented in this paper specifically address those practices that use the protocell system to directly orchestrate material processes.

As a technical system, the life-like operations of protocells require a new reading for their design relevance that examines their ordering, power relationships and agency through notions of process, complexity and nonlinearity. However, an experimental approach that directly engages matter is also needed to understand how such an approach could be technologically engaged. This is more challenging than it may first seem as, throughout the 20th century, biological systems have been likened to machines, which are object-oriented and hierarchically ordered. However, Robert Rosen [27] and Alan Turing [21] observed that the way that biological systems were represented were over-simplifications of their constituents. While the field of cybernetics introduced notions of feedback to address the dynamic qualities of life-like systems, the substrates employed shared the assumptions made by machines—that their material properties remained constant while only their relationships changed. Cultural commentator Gilbert Simondon, therefore, observed that the characteristic of cybernetic systems was in the adaptation of systems, not their transformation [28]. However, the technical program of protocells is capable of more than adaptation and may transform one kind of substance, or system, into another. The programming language that enables these instructions is based in physics and chemistry. Protocells can, therefore, be considered as a massively parallel, integrated software and hardware platform, which generates physical outcomes that are not abstracted and interpreted but entangled with their physicality and environment. The environmental sensitivity of protocells and their malleable materiality speaks to Charles Darwin’s observation that both environment and embodied program of living things shape their evolution and, as such, protocells exhibit properties associated with biological systems beyond the “mechanisms” of life, such as the ability to transform at tipping points, which may be regarded as a form of chemical punctuated equilibrium, see last row Table 3.

From an experimental perspective, the practice of chemistry was considered as a means of potentially connecting systems that are ontologically distinct. It was, therefore, viewed as a universal programming language that informs a material supercomputer which resides in the fundamental properties of atoms that is also capable of classical kinds of computation [29,30]. Recent research describes bacterial interactions as enabled by a chemical “language” with “words” [31] that can influence non-bacterial agents [32] and may even be capable of combinatorial linguistics [33]. Such findings are preliminary but suggest that sophisticated communication systems may not be exclusive to large, centrally organized brains and molecular interactions are semiotic tools for bacteria. However, the broader association between language, cognition, and bacterial communication systems is still being defined, and a great deal of further work needs to be done to verify the importance of these discoveries in this controversial field.

To establish a technical system that could provoke material transformations for design applications, an experimental model was, therefore, required that could engage with a chemical language, which could also influence the life-like chemical principles of protocells. For practical purposes, it was also necessary to select a platform that was readily observable at the human scale, as well as operating within timeframes conducive with conventional design and laboratory practices.

### 1.4. Dynamic Droplets as a Model Protocell System

**Figure 1:**
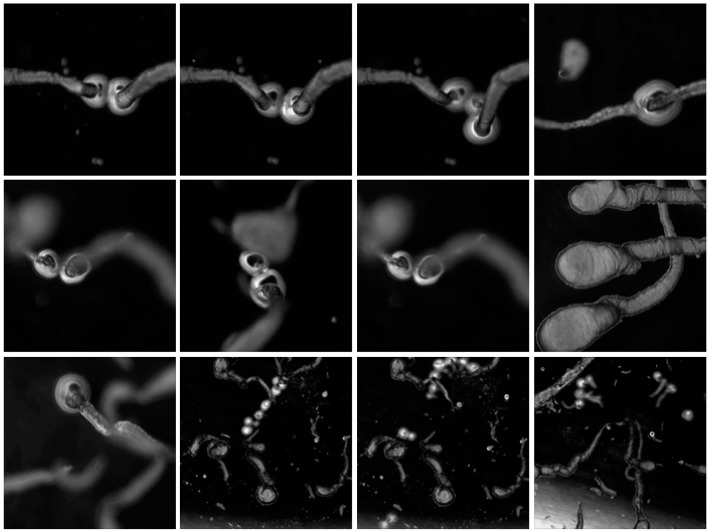
Micrographs and collage.

Note: Dynamic droplets introduce new temporal and spatial programs into microscale construction sites by spatially transforming, rather than consuming their surroundings.

Dynamic droplets are self-assembling agents that are composed from different recipes [11] but are fundamentally based on the chemistry of oil and water. They arise from a spontaneous field of self-organizing energy and can manifest as oil droplets in a water medium, or water-based droplets in an oil medium. Where oil/water interfaces occur there is a spontaneous self-assembly of molecules owing to the chemical basis for energy exchange at the droplet interface. The consequences of mass interactions are observed in the system as emergent phenomena that typically exhibit life-like behavior, such as movement. Even when the initial conditions are the same the various droplet species show a range of possible types in any given environment because of the emergence in the system, which gives rise to life-like phenomena, such as movement and environmental sensitivity, which can be observed and characterized. Dynamic droplets are influenced by internal and external factors and therefore amenable to design interventions. Dynamic droplets are restless, inherently creative agents that ceaselessly patrol and reposition their chemical networks and interactions. As dissipative structures, they throw out energy and materials to resist the decay towards equilibrium to which they will eventually succumb. This mayfly-like existence is dependent on their chemical composition and environmental context, but lasts between several seconds to many weeks. In some systems, it is possible to read the activity of a dynamic droplet through the environmental traces that are left, as microstructures and crystals that may become the site for further droplet activity, resulting in complex constructions that can be seen with the naked eye [11]. To establish a suitable model protocell system, a range of preparations including decanol/decanoate oil in water droplets [15,34] and the Bütschli water in oil droplet system were explored in a laboratory setting where it was possible to make a cursory assessment of the systems with respect to their technological potential and their suitability within a design context. The Bütschli system was examined in further detail as it consistently produced the most vigorous agents from inexpensive ingredients, see Figure 1 for a set of micrographs taken during a single experiment.

## 2. Experimental Section

A design-led experimental approach was taken to establishing the quality of performance of a model protocell system based on simple water in oil dynamic droplet system, which prioritizes the process of discovery over achieving a specific goal or answer. This approach aims to create a map of possibilities within a system, which may otherwise not have been obvious and the findings subsequently used to formalize a design solution, potentially using other methods.

Zoologist Otto Bütschli first described the dynamic droplet system used as the key model system in this series of design-led investigations, when he added a drop of strongly alkaline potash to olive oil [35].

The experimental design was modified to suit a modern laboratory and interpreted Bütschli’s original ingredients (potash and fresh olive oil) into a workable, hand-delivered system where 0.2 mL drop of 3 M sodium hydroxide was added to olive oil in a 3.5 cm diameter Petri dish, which was filled to a depth of 0.5 cm with extra virgin olive oil. These ingredients combine through a saponification reaction, in which the trigylerides of the olive oil are cleaved to produce free fatty acids and glycerol. The main ingredient of olive oil is oleic acid that constitutes around 61.09% to 72.78%, depending on the source [36]. The same brand of oil, Monini extra virgin, from Spoleto, Italy, was used exclusively in this experiment although it is not known whether different bottles came from the same production batch. All ingredients were used at room temperature.

Controls included adding a 0.2 mL drop of water to a 3.5 cm diameter petri dish filled, 0.5-cm deep with olive oil and also by adding 0.2 mL 3 M sodium hydroxide to a 3.5 cm diameter glass bottom petri dish filled, 0.5-cm deep, with canola oil (Rapeseed), from Cargill Oil Packers, which is around 85% oleic acid [37]. Systems that included a titration of sodium hydroxide were also performed.

The life-like qualities of the Bütschli system have not been formally recorded other than through Bütschli original hand drawings [35] and, therefore, required full characterization before the technological potential could be evaluated [11]. The behaviour and morphology of this system was observed and characterised in detail using a Nikon Eclipse TE2000-S inverted microscope (Tamura Corp, Escondido) with Photometrics Cascade II 512 camera and in-house software under a light microscope for approximately 300 replicate experiments under the standard conditions. Dynamic droplets were produced during a variable window of time (from 30 s to 30 min after the addition of alkaline water to the oil phase) and were photographically documented. Life-like self-organizing patterns were observed, which provided a means of introducing temporal and spatial order in the system and created a platform for further chemical programmability.

## 3. Results and Discussion

### 3.1. Observations

**Figure 2:**
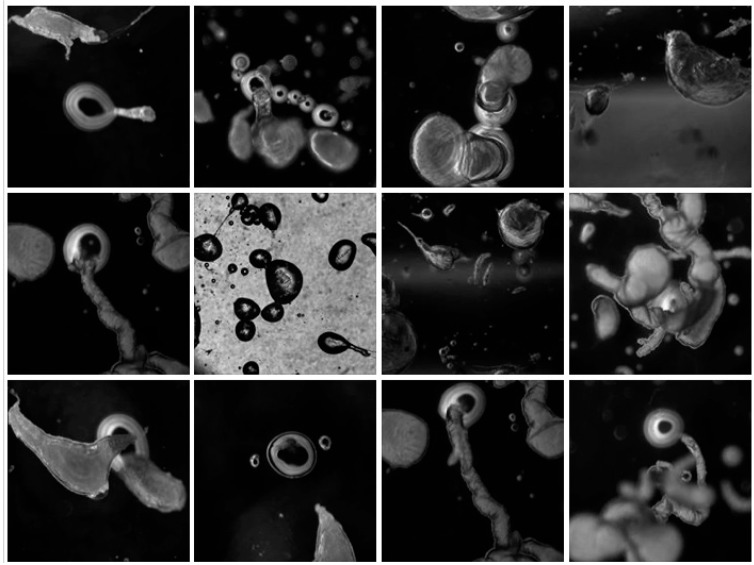
Complex structures produced by dynamic chemistries may relate to the spatial complexity produced by metabolisms, which enable the evolution of complex structures that are characteristic of organic life.

On addition to the olive oil, the strongly alkaline solution quickly broke up into organizing fronts of chemical activity and a collection of centrally placed droplets emerged from the reaction fields that were about a centimeter in diameter. The droplets were an ideal model system for observing complex material relations over short time scales that lasted up to an hour. They were inexpensive to produce, could be readily viewed at the human scale, and demonstrated life-like emergent properties. Using a modern light microscope, the droplets exhibited a range of phenomena, such as movement, group interactions, the production of structures, and environmental sensitivity, see Figure 2 for a series of micrographs depicting various interactions observed in the Bütschli system and also Table 1, Table 2 and Table 3 for an ordered presentation of series of specific findings.

From these experiments it was possible to observe that Bütschli droplets possess a set of simple operations that originate from an internal force that is conferred by its metabolism. This provides the energy that the droplets use to exert effects on their surroundings. They can move around their environment, sense it, and even produce products in parallel forms of organization and without a hierarchy of order. These spontaneous groupings make loose, reversible interactions between each other and generate the flexibility, robustness, and environmental sensitivity of the system. These interactions provide a set of morphologies that relate to the spontaneous creativity in the system but which evade simple definition.

### 3.2. Characterizing the Range of Outputs

“Osmotic growths like living things may be said to have an evolutionary existence, the analogy holding good down to the smallest detail. In their early youth, at the beginning of life, the phenomena of exchange, of growth, and of organization are very intense. As they grow older, these exchanges gradually slow down, and growth is arrested. With age the exchanges still continue, but more slowly, and these then gradually fail and are finally completely arrested. The osmotic growth is dead, and little by little it decays, losing its structure and its form” [38] (p. 151).

**Figure 3:**
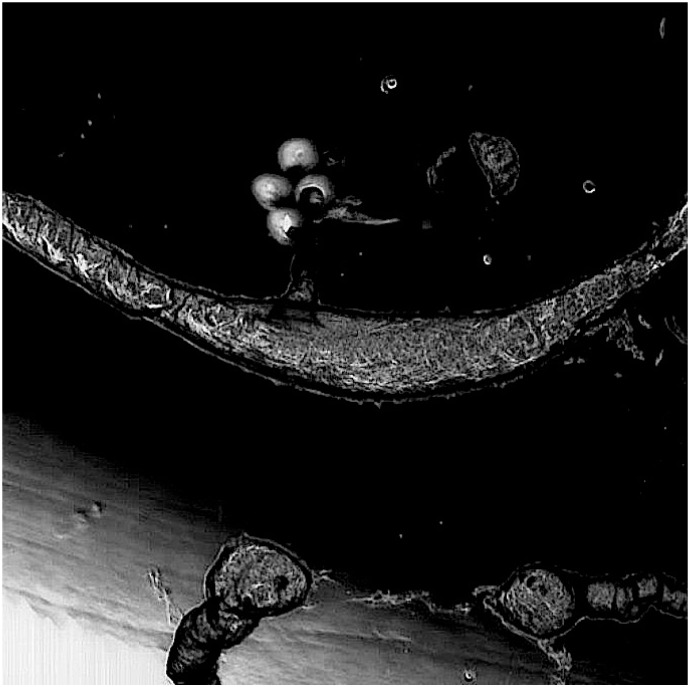
Landscape formed by osmotic structures that has been produced by dynamic droplets in an oil medium is reminiscent of the micro channels that exist between soil particles. These enable the movement of elemental infrastructures, such as air and water, through the material structure, as well as providing a large surface area for catalytic and metabolic activity.

In 1911, Stephane Leduc studied the behaviour of chemical solutions mixed together. He noted they produced strikingly life-like results that he described as “evolutionary”. Leduc likened the behaviour of these chemistries to living systems, associating the behaviour of the chemistry with terminology that is normally associated with the “life cycle” of an organism [38], see Figure 3. This section builds on Leduc’s analogy and proposes a progression of events in the Bütschli system that alludes to possible consideration and reflection on natural phenomena.

The stages of the lifespan of Bütschli droplets were organized into morphological and behavioural phases of pattern progression that occurred during three distinct stages:

- Birth (0–5 min)
- Life (30 s–30 min)
- Death (0–30 min)

Perhaps surprisingly, the behaviour of these droplets and their populations is rather conservative and predictable although these groupings operate within “limits” of possibility as they exhibit a predictable range of behaviours.

The exception to this rule is when the assemblage reaches a tipping point, where group interactions also give rise to novel, emergent, complex events that are not characteristic behaviours of any of the participating agents, but, yet, are striking and recognizable. Occasionally, a range of different phase changes in the internal condition of active Bütschli droplets takes place that may occur at the level of individual droplets, or within populations.

The findings are summarized in Table 1, Table 2, Table 3 and Table 4. A full account of the behaviour and morphology of the system is detailed in the 2013 paper by Armstrong and Hanczyc [11].

**Table 1:** Birth stages of Bütschli dynamic droplet formation.

Time after addition of alkali to oil phase	Photograph of phenomenon	Pattern Morphology	Comments
20 s	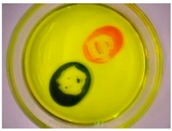	3.5-cm petri dish. Early movement dispersion of droplet and breaking up of the chemical wavefront due to changes in surface tension.	Macroscopic view of Bütschli system.
50 s	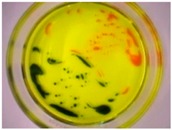	3.5-cm petri dish. Progressive movement and dispersion of droplet and breaking up of the chemical wavefront due to changes in surface tension.	Same preparation as in Figure 1 after the passage of 30 s.
8 s	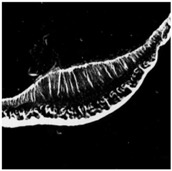	6-mm width of micrograph. Polarized field of “Fire” and “Ice”. The leading “fire” edge is facing downwards and the trailing “ice” edge is facing upwards in the micrograph.	
	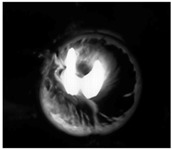	6 mm width of micrograph. Turbulent, shell-like droplets that appear as a series of sequentially emerging manifolds.	Some “shells” collapse while others self-organise into droplets with life-like properties such as, movement.

**Table 2:** Life stages of Bütschli dynamic droplet formation.

Time after addition of droplet to oil phase	Photograph of phenomenon	Pattern Morphology	Comments
2 min 30 s	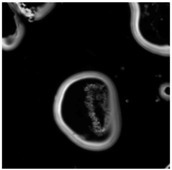	300-micron width of micrograph. Motile, droplet derived from the chaotic chemical field.	Crystalline material is visible accumulating at the oil/water interface at the posterior pole.
3 min	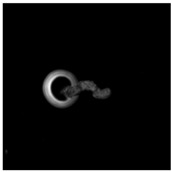	6-mm width of micrograph. Droplet with osmotic crystalline deposit.	Crystalline material is visible as an osmotic microstructure attached to the droplet at its posterior pole.
8 min	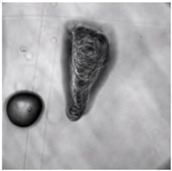	300-micron width of each micrograph Osmotic structure seen with and without fluoroscopy in which the Bütschli droplet has just detached from an osmotic structure.	These figures are of the same structure.
	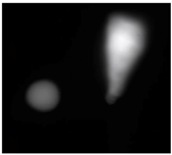		
10 min	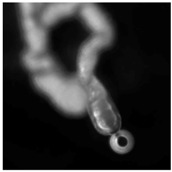	6-mm width of micrograph. Bütschli droplets produce deposits of sodium oleate at the trailing end of the motile droplet where they accumulate and extend to form fluid-filled microstructures.	
2 min	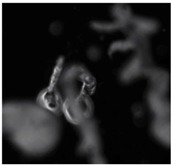	6mm width of micrograph. Bütschli droplets before fusion.	Fusion events are spontaneous and may be the generative agency for the production of compound, complex, osmotic micro structures.

**Table 3:** Death stages of Bütschli dynamic droplet formation.

Time after addition of droplet to oil phase	Photograph of phenomenon	Pattern Morphology	Comments
8 min	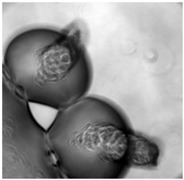	300-micron width of micrograph. Two Bütschli droplets engage active interfaces generating various, dynamic points of contact. They continue to make contact until the product (sodium oleate crystals) obstructs the interface between them.	Interfaces between droplets persistently osculate.
12 min	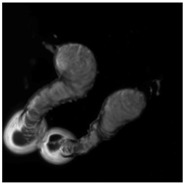	6-mm width of micrograph. Bütschli droplets “mirroring” one another.	
12 min	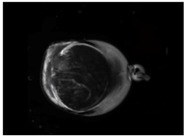	6-mm width of micrograph. A smaller Bütschli droplet is interfacing with a much larger one.	The droplets remain in close proximity with each other until the build up of soap crystals occludes the oil/water interface.
8min	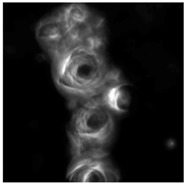	6-mm width of micrograph. Bütschli droplets in a simple chain formation.	Periodic oscillations are observed in agents during a chain-forming event.
10min	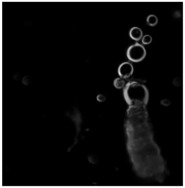	6 mm width of micrograph. Bütschli droplets in a complex chain formation.	“Protocell roses”.
15min	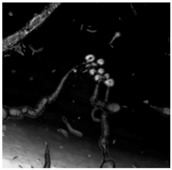	6-mm width of micrograph. Two droplet assemblages merge and suddenly change behaviour and morphology.	Phase change behaviour observed during the formation of an assemblage when a “tipping” point is reached. Such events were observed on separate occasions.
	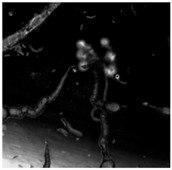		

**Table 4:** Modified Bütschli droplets.

Time after addition of droplet to oil phase	Photograph of phenomenon	Pattern Morphology	Comments
20 min	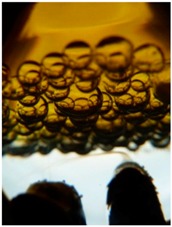	300 micron width of micrograph. Fine crystals of sodium oleate are accumulating at the oil/water interface.	Crystal deposits accrue at the “posterior” pole of the droplet.

### 3.3. Ontological and Epistemological Issues Raised by Bütschli Droplets

Owing to the continually changing nature of the Bütschli droplet outlets, ways of classifying events within the system were considered. Each droplet possesses a set of properties that are not fixed but are continually transforming themselves through interactions between themselves and their environment. These exchanges also create the conditions in which further populations of droplets move through and perform within the reaction field.

The Bütschli system potentially offers a technological platform that exhibits non-classical behaviours, which invoke a distinct set of concepts that are different to those of classical technical systems. While Bütschli droplets may be framed within the language of process philosophy and scientifically characterized through the principles of complexity, observing the system is inevitably mired in established deterministic concepts and aesthetic expectations [39]. This makes it difficult to view and describe the constantly changing Bütschli system without trying to define its performance within pre-existing knowledge sets. However, this is exactly what needs to be done if the full potential of this emerging technology is to be fully explored and imagined. Indeed, the Bütschli system may yet prove to be “post-epistemological”, or unclassifiable in any coherent, meaningful way using traditional modes of classification, such as the system proposed by Carl Linnæus [40].

Although man-made, and in that sense “artificial”, the life-like performance of the Bütschli system provides an opportunity to consider the emergent characteristics as a subset of living qualities in order to construct a more thorough understanding of the system as a whole. However, there is no classification system to characterize dynamic life-like chemistries. Linnæus imposed an order on natural systems, which included three domains: animal, vegetable and mineral that therefore embraced both living and non-living materials and facilitated a comparative understanding of these systems by appreciating similarities and differences [41]. Of interest is Linnæus’ taxonomy of stones, which he some of the properties of living things. In particular, Linnaeus suggested that rocks grew by way of an accretion process, such as when sand aggregated and became sandstone or, when the apparent clumping of clay particles formed limestone. He also included the formation of quartz in his classification system, which he proposed was due to a “parasitic” mechanism. However, minerals were dropped from taxonomic classification during the eighteenth century and are absent from Lamarck’s 1809 classification scheme “Zoological Philosophy” [42], which focuses exclusively on the cataloguing of animals. Additionally Ernst Haeckel’s famous 1866 “Tree of Life” [43], based on Charles Darwin’s taxonomic diagram [44], equated phylogeny with the story of evolution and excluded the mineral world from phylogenetic ordering systems. It is possible that the omission of minerals from a scientific ordering of the natural world may also have been, at least in part, influenced by the popularization of Louis Pasteur’s germ theory [45], which refuted a widespread belief in spontaneous generation, where life was thought to be created directly from inert matter [11].

The approach taken in reporting the observations is relevant to current systems of classification used in biology and natural history, which may help relate non-living phenomena to biological systems through a description of their pattern morphology. There is much to be learned through comparative analysis and my research attempts not only to observe, but also to construct an understanding of the characteristic of the life-like properties of the Bütschli system as the basis for further study.

Conventionally, dynamic systems are described by recognizing geometric domains, such as patterns and metapatterns. However, there are semantic problems with such an approach since pattern-recognition, through identifying particular kinds of morphology, reveals nothing about the process of production, which is closer to an algorithm that represents a set of rules than any particular geometry and only encapsulates one particular time frame in a sequence of events. For example, very similar patterns may be generated within different media, such as DNA producing mollusk shells (physical systems) and the graphical modelling of shell-like structures on a computer screen (virtual systems) [46].

**Figure 4:**
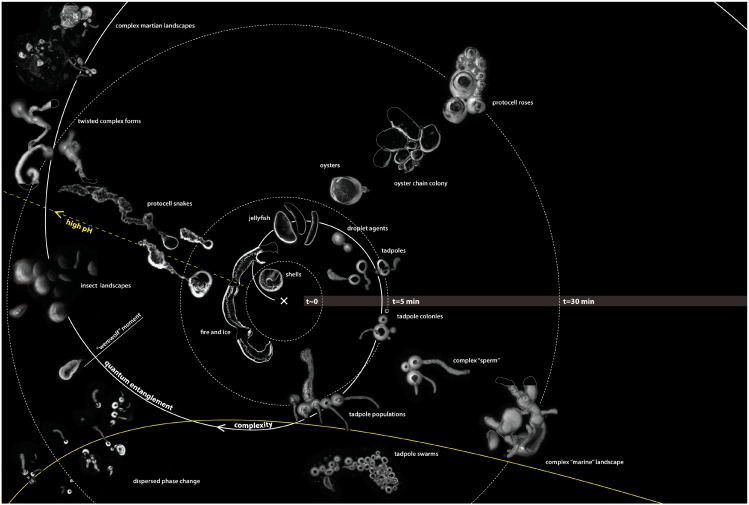
This diagram depicts dynamic droplets as “actors” that operate within the many variable influences encountered in their oil field as an ontolological “map” of events. While the diagram is drawn as a 2D topology, the possible events within the field are manifold and open up multi dimensional spaces through their interactions with, continuous, multiple contingencies that shape the evolution of the system.

The Bütschli system may be analyzed through the lens of process philosophy to establish possible reference points for evaluating the performance and development of a nonlinear technical system. Matt Lee uses the term “oceanic ontology”, which is an assessment system that produces maps rather than theories of concepts [47] (p. 27) as a way of reading transformational events within nonlinear fields of action. To test this approach, an oceanic ontology of the Bütschli system was produced that drew on observations from the 300 replicate Bütschli experiments and subjectively mapped specific relationships using exploratory graphical approaches, see Figure 4.

**Figure 5:**
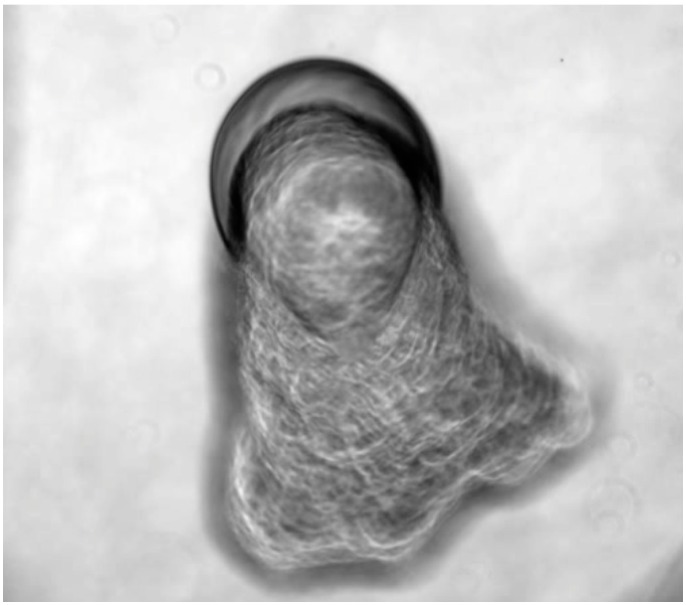
Oyster-like, thick, osmotic structure produced by dynamic droplets.

The diagram is centred at time zero, from which concentric circles radiate, representing an exponentially increasing series of time intervals. This logarithmically increasing function proposes to encapsulate the intense self-organizing activity that happens early on in the chemical reaction and falls off rapidly with the passage of time. An estimated ninety percent of chemical activity is completed within five minutes of activation of the system, although individual droplets have been observed to be active as long as an hour after their genesis. A spiral that represents complexity that also radiates from the origin and depicts the high frequency of events around the start of the reaction that becomes less frequent as time unfolds. The various morphologies and behaviours that indicate change in the system are subjectively grouped according to the authors’ experimental findings and interpretations. For example, the complex oyster chains are distinct in appearance but only differ by degree, from the complex marine landscapes. Specifically, “oysters” produce a large mass of material and their soft bodies bulge from their material shell-like tethers, which anchor them, as shown in Figure 5.

**Figure 6:**
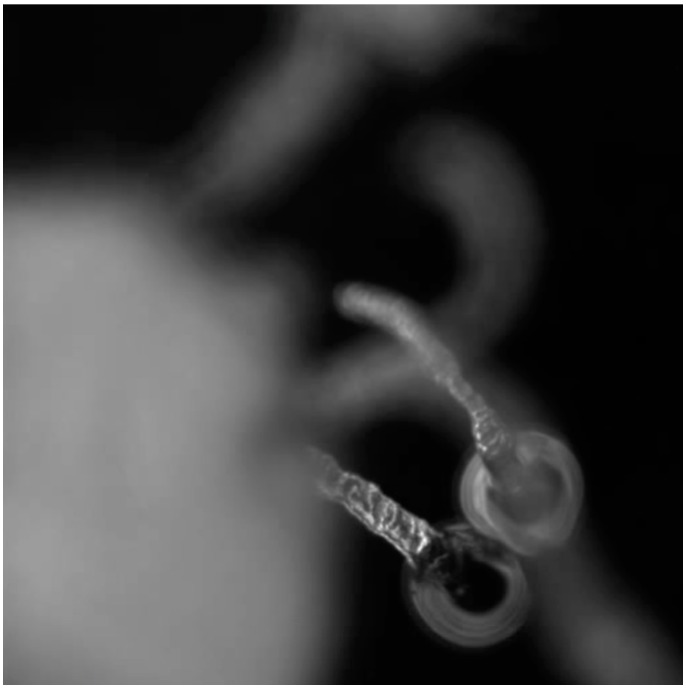
Thick, osmotic structures being produced by dynamic droplets that are moving away from their deposits and producing “marine landscapes”.

In contrast, “marine landscapes” are composed of a variety of largely inert forms that have been produced by droplets that would previously have been described as “oysters”. However, the undulating droplets are long gone leaving only a trail of residues behind them, as shown in Figure 6.

The diagram also indicates the impact of chance events from a source external to the system as an incidental trajectory that intersects with the fundamental progressive vectors of the Bütschli system. It represents disturbances in the environment, such as changes in ambient temperature, or physical disturbances. This external vector also touches the spiral of complexity and in this case, implies that in this specific context, agents within the system could reach tipping points.

However, while attempting to generate relationships between events, the diagram is not empirical but makes use of graphical qualities and metaphor to convey complex occurrences within the reaction field. For example, the “werewolf moment” is characterized by extreme droplet agitation and the rapid production of residue, which bestows it with a rather “hairy” appearance. This striking event is most likely precipitated by the ratio between droplet surface area and the volume of the droplet that are optimized and therefore rapidly consume the dynamic agent. The rapid precipitation of product over the droplet surface causes drag that precipitates erratic movement. This excitement phase typically lasts for around a few minutes and produces a large amount of residue. This is swept to the posterior end of the droplet by molecular action and physical forces, where it is suggestive of a “tail” that exerts a great deal of drag on the system. This series of complex events immediately precede droplet inertia as the dense precipitation extinguishes the droplet metabolism by completely occluding the interface. The werewolf moment is, therefore, a pre-terminal event for the droplet, since its outcome is quiescence.

The Bütschli system represents an active chemical body that is at far from equilibrium and shaped by its context. It possesses distinctive material programs that confer the system with recognizable characteristics and patterns of behaviour, which exist within a silent network of material systems that are conjured when substances are mixed. Such phenomena resist empirical definitions and court a post-epistemological status, which questions existing categories and identities that take place within much broader field of events. Indeed, this realm has become so naturalized that it has receded into the background of our daily lives as a matrix of constant production, which Bruno Latour refers to as OOWWAAB (Out Of Which We Are All Born) [40]. Of course, this dynamic field of chemical transformations is culturally recognized as Nature, and embodies a vast horizontal plane of making, from which life on Earth has sprung.

In the natural realm boundary interactions are not exclusive to the behaviour of populations, such as flocks of migrating birds, schools of dolphins, or dynamic droplet assemblages, but also exist as strong and weak forces between objects including gravity, electromagnetism, strong and weak nuclear forces. These generate a host of interactions including, attractions, repulsions, amplifications and extinctions that may be observed at the interface of trembling dynamic droplets, which are infused by the medium in which they exist to exert effects on the ordering of material relationships. This creates a synthetic scenario that builds new groupings, identities and forms of order that constantly propose new networks of operations through which living systems may constantly adapt and evolve.

### 3.4. How Might Protocells Be Applied Beyond a Laboratory Context?

**Figure 7:**
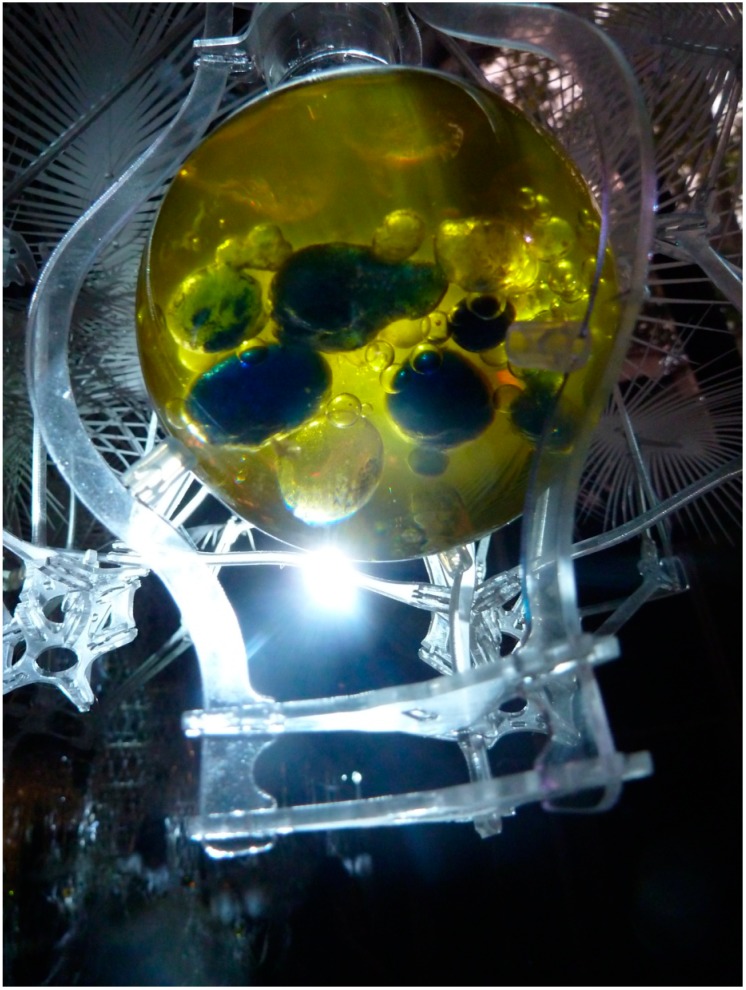
Modified Bütschli droplets respond to environmental conditions in flasks that are open to the air. Incubator Flasks were suspended in the Hylozoic Ground matrix and positioned over light emitting diodes (LEDs) to capture heat and light from the activated cybernetic matrix.

For the protocell to useable as a technical system, it must be possible to shape its operations. The protocell itself may be as a “leaky” body that is responsive to control strategies that that operate through natural computing techniques to open up new design and engineering possibilities. The designer therefore establishes the selective permeability of protocell agents and sets limits for their potential interactions. For example, internal conditions can be manipulated by adding a soluble mineral, such as Copper II Sulphate, to an attenuated droplet to produce green copper carbonate precipitate in the presence of carbon dioxide. Additionally, introducing organic solvents to the olive oil medium, such as ethanol and acetone, reduce surface tension and cause a rapid mass movement of droplets towards the source. However, for natural computing to have cultural relevance the systems must exist at a scale by which they can be observed, manipulated and inhabited. By adding an inhibitor from the products of the reaction itself, the selective permeability of the agents is greatly reduced and droplet interactions can be slowed down and their diameter increased from around a millimeter up to several centimetres in diameter. Modified Bütschli droplets can even be applied at an architectural scale in the most pragmatic sense, by converging their interactions with other systems. In the “Hylozoic Ground” cybernetic installation designed by architect Philip Beesley for the 2010 Venice Architecture Biennale, modified Bütschli droplets notionally operated as a giant “smell” or “taste” system that responded to exhaled carbon dioxide from gallery visitors that dissolved into open flasks and produced brightly coloured carbonate precipitates on contact with soluble nickel II, iron II, iron III, and cobalt II salts, see Figure 7.

**Figure 8:**
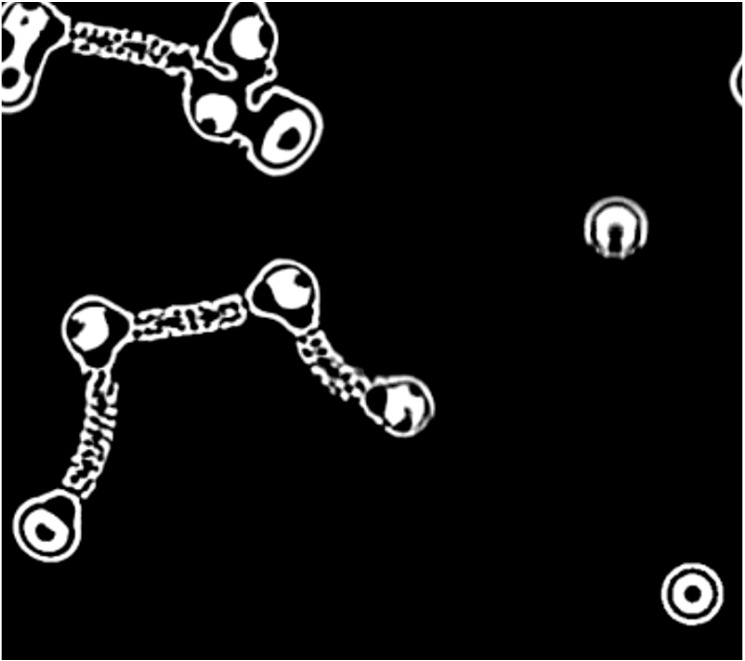
This version of Conway’s Game of Life has striking homologies with the Bütschli droplet system in that agents are producing self-evolving traces in their environment.

Dynamic droplets achieve their striking outcomes by spatializing chemical reactions and effectively inserting time and space into matter. This appears to enable the technical system to evade decay towards equilibrium [48] and is consistent with biological systems that are highly reticular and are spatially and temporally organized. For example, the endoplasmic reticulum catalyzes a host of metabolic processes and allows substances to be transformed so they may be the end produced of one reaction and the beginning of another. In this way living systems maintain their operational integrity as transformers, as not all their chemical relationships are simultaneously active or in the same place, which creates an opportunity for the formation of new chemical interactions and transitional states. While no two systems are exactly alike, they display consistent patterns and behaviours. This provides opportunities to develop theoretical models of their interactions, so that their technical qualities may be developed further. Digital modeling of Bütschli droplets has been explored with Carlos Olguin and his Bio/Nano/Programmable Matter Group at Autodesk. However, the range of types produced by an algorithm approach has so far been very limited in comparison with the diversity of forms produced by the actual system. An incidental observation, where homologies between populations of droplet interactions were noted with Stephan Rafler’s Smooth Life [49], a version of John Conway’s Game of Life [50], see Figure 8. Although the digital model exhibited many similarities in terms of pattern generation, it did not, however, exhibit the striking phase changes noted in the chemical system.

### 3.5. What Design Challenges Does the Protocell Technical Platform Pose?

Since protocells are context sensitive in their performance, many local challenges with a given system are likely to arise that would typically need to be dealt within the context of a specific project. However, fundamental challenges must also be addressed when preparing to work with a technical system that can be viewed as operating differently than machines. A set of concepts and ideas that most appropriately describe the process-philosophy based order and possibilities provided by the new technical system have been provided. These are compared with the ontology of machines to establish a portfolio of concepts that frame an approach towards working with protocells, which may also be regarded as a particular species of natural computer, see Table 5. However, this is not an exhaustive list.

**Table 5:** A comparison between machines and natural computers.

Technical constituents of machines and protocells		
	Machine	Natural Computer/Protocell
Component	Object	Agent
Order	Series	Parallel
Power structure	Hierarchical system	Non-hierarchical
Functional system	Machine	Assemblage
Energy	Extrinsic	Intrinsic and extrinsic—spontaneous operations may be prolonged with resource supply.
Control	Hard	Soft
Transformation	Binary—on/off	Variable states. Generally conservative but may behave unpredictably and collapse or transform at tipping points
Points of influence	Internal	Internal and external (environmentally sensitive)
Ability to form networks	Insular. Creates barriers between systems	Facilitates technological convergence by building networks of exchange

While machines may be thought of as deterministic systems that are internally driven and discretely bounded, natural computers are leaky, open systems that are environmentally sensitive, stochastic and more difficult to establish formal principles for their applications. However, several specific concepts related to expectations in the performance of protocells as a technical system warrant further consideration, namely:

(a) Assemblage
(b) Soft control and agency
(c) Infrastructure

### 3.6. Assemblage

The term “assemblage” refers to meta-bodies of non-hierarchically ordered, participating agents—or empowered objects [51] (p. 23)—that actively forge relationships between each other. However, from a technical perspective, the concepts underpinning the term also embody the operating system present in natural computing systems. From a technical perspective, assemblages function within a complex material realm that is far from equilibrium. They are able to forge relationships between different kinds of bodies or systems, establish material networks and even transform groupings of agents from one state to another. Potentially, the assemblage constitutes a powerful operating system that may even enable us to make a transition from an industrial to an ecological culture.

### 3.7. Soft Control and Agency

Far from equilibrium states, natural computers engage in parallel forms of multi dimensional processing. Such systems invite “soft” forms of control [52] since their agency is distributed throughout their constituent assemblages. The multiple agencies within an assemblage give rise to organizing centres that may generate propagating waves of activity, which attract, activate, and inhibit interactions within a given system. Technical systems, such as protocells, therefore, require soft control strategies, whose outcomes may be choreographed, rather than instructed. Although soft control strategies may seem impossible to realize, in practice they have been used as part of the portfolio of creative disciplines, such as gardening, painting and cooking. However, soft control systems do present challenges when viewed through traditional engineering approaches, such as in soft robotics, which uses materials with nonlinear properties to “simplify” computation [53]. However, observing the challenge through the lens of process philosophy, lively materials do not fundamentally change the complexity of a challenge. Rather, they spread the agency of the potential resolution of the system through many bodies. Such concepts have not been formalized within the field of robotics and, therefore, it is difficult to formulate a coherent strategy towards working with novel materials, although this field is rapidly evolving. While nonlinear dynamics and complex systems are far from being mathematically resolved, researchers, such as Albert László Barabási, are providing clues into how complex dynamic fields may be strategically influenced. For example, it is better to find sites for intervention a distance away from important organizing hubs to alter the performance of a complex system, since powerful organizing centres tend to be so robust that they dampen down disturbances and are resistant to perturbations in the system [54].

### 3.8. Infrastructure

Since protocells are embedded in their environment, their performance is contingent on their context, as well as the flow of energy and matter through their environmental niche that are delivered by elemental systems, or the infrastructures of life (air, water, heat, earth). However, in a broader context, modern spaces are designed around machine performance, the great architect Le Corbusier famously observing that buildings are machines for living in. While energy and data provision are essential for the smooth running of machines, a different set of infrastructural requirements need to be met if natural computing systems are to be widely adopted as design and engineering strategies. Indeed, infrastructural design may not only be essential in developing protocell-based applications but may even be critical for the transition from non-life, to living to being fully alive.

Contemporary approaches taken towards protocell design regard them as componentized objects, which have particular requirements to meet the minimum conditions for “life”, such as compartment, metabolism, and information [16]. The assumption being that when some cumulative threshold is reached that the protocell model will be granted the status of being truly alive. However, by taking a componentized, object-centred view of artificial agent developers encounter difficulties, when life is not defined by the acquisition of life-like components alone, but also engage with existential criteria, which are invoked in Alan Turing’s Imitation Game [23]. In other words, when “life” is not exclusively defined by materiality of the system, but also from a philosophical and ontological positions, then adopting non-mechanical approach to the production of life could produce robust life-like systems that could provoke recognition as being fully alive in other ways.

For example, perhaps one criterion for success in the production of a living system would be to provoke a stable dissipative structure. Such a body is not componentized but would spontaneously arise when under certain conditions energy and matter continually flow through a space. From a technical perspective, such simple structures are exemplified by the Bütschli system, which are not technically alive, but do persistently exhibit strikingly life-like behaviours that can be shaped by natural computing techniques to usefully complete tasks, such as site-specific precipitation. Potentially then, creating the preconditions for life, as opposed to building life-objects, is another approach that could be used to construct protocells. Already technical systems exist that can generate artificial tornadoes and whirlpools [55], and experimentally hypercycles [56] and continuous flow systems [57] prolong dynamic reaction times and may prove useful in increasing the probability of life-like events in a space, as well as being instruments that could shape the performance of these systems, so that they may be used effectively as a technical platform.

### 3.9. Technological Convergence

The performance of protocells does not have to be constrained by the limits of its own platform, but may also be extended by combing its operations with other technological platforms such as 3D printing, which can further increase the possibility of developing radically new techniques and technologies that may take on a range of different appearances from something as mundane as a set of chambers in which reactive chemistries can explore new, complex configurations, to wholly synthetic environments.

In the multidisciplinary “Wet” Fab, or wet fabrication, event held at the Cronin Lab in Glasgow, and funded by the Engineering and Physical Sciences Research Council (EPSRC) designers, computer scientists, engineers, physicists and chemists explored the Fab@Home platform together to “*produce some new science*” [58]. During the two-day event, dynamic chemistries were spatially positioned using a Fab@Home printer. While the tests from the workshop were exploratory, introducing inorganic salts into gels and oils, the Cronin group went on to develop a prototype system that could sequentially build complex chemistries [59]. The chemical “reactionware” and the reagents themselves were made using the printer. The reactionware itself was fabricated using bathroom sealant to create a series of reaction chambers with precisely specified dimensions, which were connected with tubes of different lengths and diameters. Once the container system was developed, then a range of different chemistries could then printed into the chambers to generate a reaction sequence that produced increasingly complex molecules. The “Wet” Fab system, therefore, combines the notion of a reactor with a reaction. The printer technology not only orchestrates the chemical sequences but also shapes the environment in which they are formed. This is a qualitatively different approach to the neutral environments in which chemistry is usually conducted. Future developments may explore printing catalysts into the wall of the reaction chambers, or even ultimately create complex biological simulations using printed tissue cultures to simulate chemical reactions taking place within the body. Indeed, this reactionware model could transform the process of drug discovery and testing by being able to quickly and cost-effectively screen the effects of new molecular combinations. Should the platform be productized as a miniature laboratory where inkjet cartridges may be supplied with different chemistries and software so that the printer can fabricate the right containers and chemical sequences, it may function as a portable laboratory that could for example, greatly increase access to medicines and the ability to manufacture new formulations. Indeed, “Wet” Fab changes the authorship of the reactions where the chemistries themselves possess agency within differentiated environments and directly contribute to the innovation process [60].

### 3.10. Examples of Protocell Applications Beyond the Laboratory

A series of design-led explorations through architectural projects have led to a range of experiments, models, prototypes and speculative applications for protocell technology in an architectural context. The overarching goal of these investigations is to explore the possibility of producing “ecologies for thriving in”, which would potentially signify an ecological age in architectural design that could potentially have life-promoting qualities—and would replace Corbusier’s modern industrial doctrine of “machines for living in” [61], which, at best, has a neutral effect on our environmental systems.

#### 3.10.1. Hylozoic Ground Chemistries

**Figure 9:**
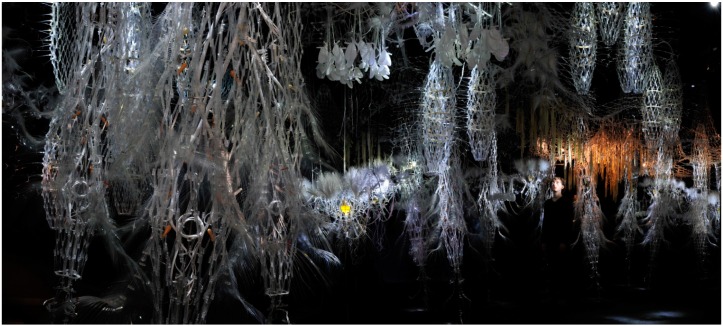
Hylozoic Ground installation, Canadian Pavilion, Venice is a cybernetic matrix that integrates a range of different “organ” and “tissue” types, such as swallowing tubes (tapered cylindrical structures to the right of the photograph) and sound organs (clustered leaf-like structures in the centre of the photograph). The challenge was to design a set of dynamic chemistries that would aesthetically and functionally complement the soft mechanical systems. A centrally placed (yellow) chemical organ can be seen centre field.

The Hylozoic Ground is an installation by architect Philip Beesley, which was Canada’s national entry for the Venice 2010 Architecture Biennale [62]. The Hylozoic Ground installation consists of a cybernetic matrix with a primitive neural network and sensory actuators that allowed the system to interact with a gallery going public. It provided an evolving experimental platform that visualized and shaped the material connectivity, novelty, and transformation that already takes place within natural systems (represented by the gallery environment) and coupled them with artificial systems (the cybernetic matrix). A series of dynamic chemistries were designed to connect the cybernetic matrix and gallery through a different kind of interface that enabled material transformations to take place over the course of the three-month installation. These Hylozoic Ground chemistries served as natural computing models that could respond morphologically, chemically and poetically to the installation’s themes and programs, specifically by responding to the expired carbon dioxide of the gallery visitors and its accumulation within the installation site. The work explored notions of “life”, ecology, the quality of spatial experience, and dynamic systems. The question of scale was implicit in this design challenge [63,64,65], and, for example, the millimetre scale field of action of Bütschli system could be further increased to the metre scale. This was achieved both by slowing down the metabolism of the droplets and also by entangling distributed populations of the chemistry in flasks within the cybernetic matrix. Dynamic chemistries enabled artificial and natural agents to form notional ecological networks within the gallery space that included installation, audience, and environment, see Figure 9. These exchanges took place through the hardware of dynamic chemical objects and metabolic software that enabled the deliberate modification of these networks by the range of actors within the system. While the underlying metabolic systems are sufficiently robust to accommodate local disturbances, the redistribution of chemical flows within these networks was poetically proposed to possess the potential for producing new kinds of Nature, whereby the synthetic ecological exchanges were poetically likened to the material complexification and diversity that shapes the proto-ecologies of natural systems. These arise from an existential sea of deeply entangled, mutable, transitional, and ambiguous agents that are subjected to selection forces, which enable some configurations, persist, transforms others, and cause many to wither.

#### 3.10.2. Future Venice

Future Venice is an architectural project that imagines an alternative sustainable future for the city by growing a synthetic reef under its foundations. It is experimentally developed through the actions of a range of modified dynamic droplets, which act as an urban scale natural computer.

The droplets are designed to move away from the light and use dissolved minerals and carbon dioxide when at rest, to collectively produce a kind of “biocrete”. Droplet titration as required, is used to “grow” the structure by adding aliquots of self-assembling mixture to the light soaked waterways of the city. Once in the canals, the droplets are programmed to move towards the darkened foundations that stand of narrow woodpiles and gradually produce a biocrete accretion technology. This spread the weight of the city over a much broader base and attenuates the city from sinking into the soft delta soils on which it was founded [66] (pp. 72–73).

**Figure 10:**
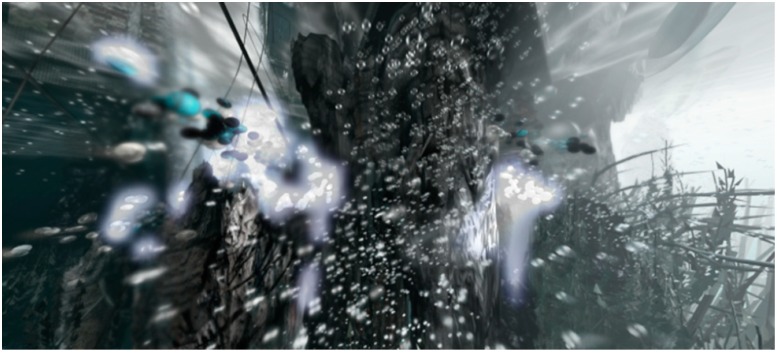
View underneath Venice’s foundations that stand upon woodpiles demonstrating the potential action of a city scale morphological computer composed of smart, programmable droplets. This speculative assemblage-based technology proposes to harness the collective action of light sensitive droplets that are programmed to move towards the darkened foundations of the city to grow an artificial limestone reef. This structure aims to gradually spread the point load of the city over a much broader base than offered by the narrow woodpiles as well as providing new ecological niches for the marine wildlife.

**Figure 11:**
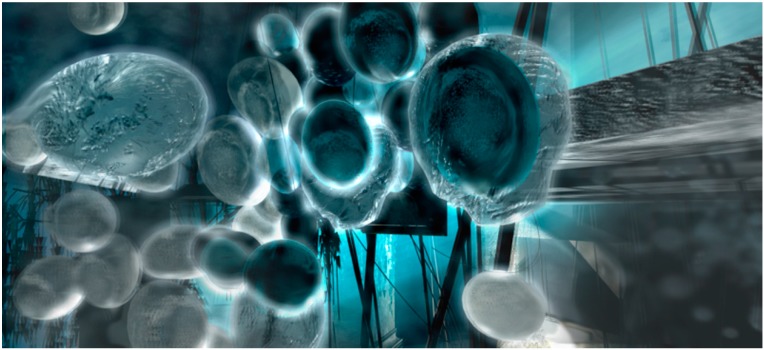
View of programmable droplets coming to rest underneath Venice’s foundations as their light sensitive metabolism reaches equilibrium and activates a second metabolic process that enables the droplets to use local minerals and dissolved carbon dioxide to grow a solid, reef-like structure.

**Figure 12:**
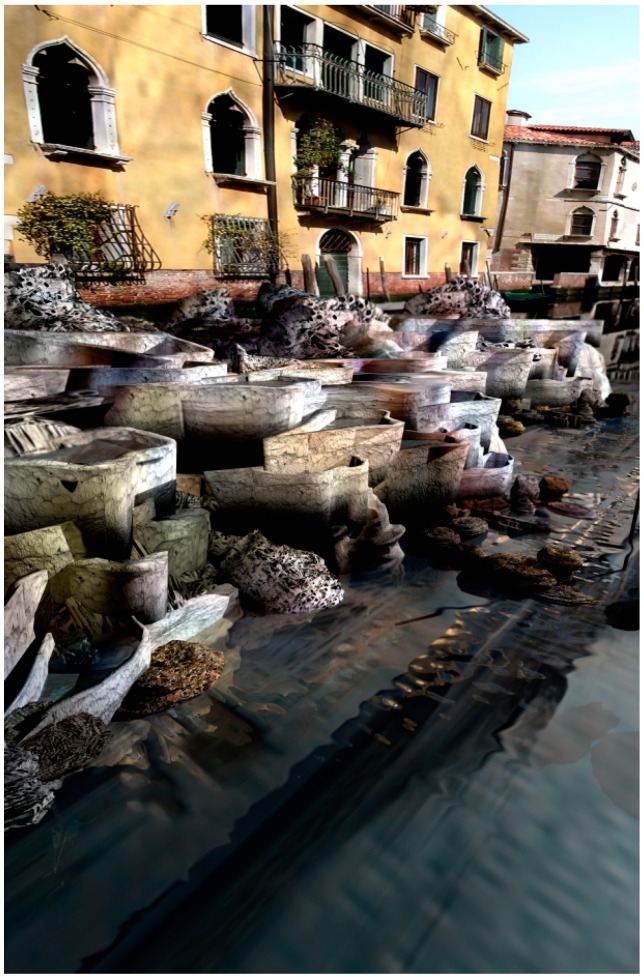
Droplet assemblages, which grow the structural and physiological framework of an artificial reef under the city, may produce porous, pumice-like formations over time that reflect mineral compositions through their coloured layers, like stromatolites. The resultant architecture does not obey a top-down paradigm but is codesigned through the entangled metabolic interactions of marine and human populations.

**Figure 13:**
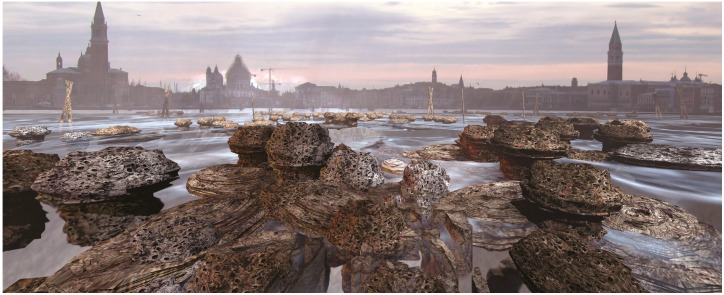
Stromatolite-like formations in the Venetian canals are shaped over time through the multiple interactions of heterogeneous actants and assemblages to produce mineralised material. This process already takes place within the waterways of Venice. An assemblage-based droplet technology is anticipated to augment this process. The collective action of programmable, life-like chemistries may produce thrombolites within the shallow waters of the Venice lagoon.

Of note, the marine organisms in the waterways already produce a kind of biocrete and it is anticipated that the protocell system will work with the marine animals to co-construct an architecture that is meaningful to both the creatures of the lagoon, as well as the city inhabitants. Should the environmental conditions of Venice change and the city dry out rather than drowns as currently predicted, then the system could potentially change the range of its outputs. Rather than growing sideways to spread the minerals over a broad base, the accretion-producing droplets may deposit their material on the woodpiles, sealing them from the air and stopping them from rotting, see Figure 10, Figure 11, Figure 12 and Figure 13.

Venice is an ideal site for exploring the theoretical potential of natural computers as an architectural technology, since the watery foundations create the conditions in which matter and energy can freely flow around the site. Indeed, a simple model of this protocell system was tested in tanks of Venetian water on the lagoonside with architecture students from the University of Venice. A simple droplet recipe using a clear oil (Diethyl phenyl phalate) of specific density of 1.12 g/cm^3^ at 20 °C, into which calcium II sulphate crystals were ground into a paste was designed to produce mineralizing “shells” on contact with the dissolved carbon dioxide in the lagoon water. Currents were generated in the tank through direct agitation to test the robustness of the system, to which the soft mineral bodies successfully proved robust and reassembled, which was an essential attribute for this platform to survive within the Venetian waterways. Infrastructure was also essential in these design-led experiments to optimize the performance of the protocells. The aqueous environment enabled the droplets to exhibit their life-like qualities, facilitated their movement in the environment and also removed waste products. These early explorations suggest that protocells may be meaningfully applied within an architectural system with significant impact. Potentially, a life-like technical platform may change the goals of a building, which is no longer an inert site for machines but an active interface that could catalyse the continual flow of materials through a site. In other words, with the advent of protocell technologies, buildings could potentially become life promoting, not just for humans, but also for entire local ecologies.

#### 3.10.3. Living Buildings

Since technology is most concentrated in urban locations, the impacts of new technological platforms are likely to be most intensely experienced in our cities. New life-like technical platforms, such as protocells, may enable buildings to make an origins of life style transition from being inert surfaces, to becoming lively interfaces that can act in our interests. Such a process could be facilitated through the design of architectural “organs”, which are spatially imagined as hubs of bio/chemical activity, flow and transformation within a site. From a pragmatic perspective, architectural organ systems are likely to be specialized aquariums that contain microcellular organisms, such as bacteria and algae, or even smart chemistries, like protocells. Architectural organs would then house these active communities of agents and support them by providing infrastructures and media that enable them to work in equivalent ways to machines, such as producing heat, filtering water or fixing carbon dioxide. By feeding the metabolic processes within these tanks with “food” or nutrients, such as carbon dioxide, organic waste or grey water, the many bioprocesses within these micro agricultures would be free to process and exchange matter. Such organ systems could be engineered in ways that rendered them invisible to residents by situating them in under-imagined sites within our buildings, such as under floors or in cavity wall spaces instead of inert insulation. They could also be highly visible, such as in Phillips Microbial home [67]—where voluptuously shaped bio processors transform waste products into useful substances within a locally defined ecology. For example, food waste could be turned into biogas and compost to feed new plants and provide energy for the home, or even commercial buildings [68]. Strategically positioned, these architectural organs may be connected into networked activities that could potentially give rise to buildings with discrete physiologies that strengthen the material exchanges within a community through networks of metabolic processes. Such technologies would ultimately change the environmental performance of building from the construction of sites that minimal interact with environments through resource and energy conservation into places that act as biotic, life promoting oases for human and nonhuman communities.

**Figure 14:**
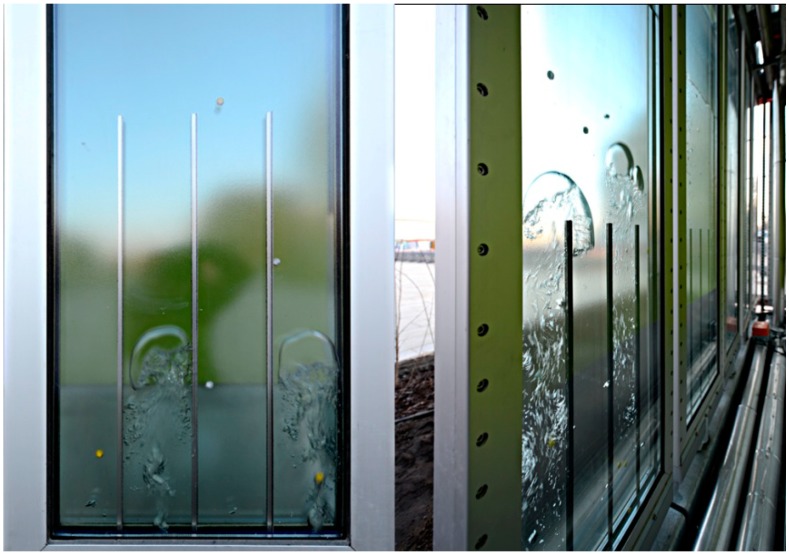
This façade designed for the International Building Association in Hamburg houses green microalgae, which produce biomass from carbon dioxide and sunlight. This product is collected and in turn, is used to create heat for the building.

However, such notions are not entirely speculative. The construction-engineering cooperative Arup is currently evaluating facades that house algae to provide energy for buildings in a project designed for the International Building Association in Hamburg, see Figure 14. These algae facades are essentially aquariums full of microalgae that are fed by sunlight within tall, narrow glass panels through which carbon dioxide is bubbled. As they flourish, the biomass of these microorganisms is syphoned off, dried, and combusted to offset energy consumption within the building [69].

Indeed, chemical and biological assemblages may even be increasingly used in the production of traditional architectural materials by growing them, such as Philip Ross’ furniture produced by fungal roots [70] and Ginger Krieg Dosier’s “Bio-bricks”, which are made from bacterially fused sand particles [66] (p.113). In the near future these materials will be used on a larger scale and will double up as both material and technology taking the form of living construction systems. For example, Magnus Larsson’s architectural projects “Dune” proposes that a giant bacterial biofilm could function as an organic “printer” and potentially reverse desertification, by traveling through the desert and creating solid ground for the settlement of plants and other organisms [66] (pp. 62–65).

### 3.11. Implications for Designing with Protocells

Designing with protocells not only invokes the direct engagement with environmental conditions but also establishes a technical relationship with Nature. From an operational perspective metabolic systems are harnessed to perform useful work, where substrates are not denatured and consumed, but transformed and even integrated into new bio/chemical networks that make up the natural world. For example, the technologies of gene modification enable organisms to make entirely novel substances like the mass production of spider silk protein from goat’s milk [71]. While many of these natural technologies are being assimilated back into industrial systems as “soft” machines, like yeast within a brewery, their full portfolio of impacts is being explored in the emerging scientific fields of living technology [72], synthetic biology [73], the origins of life sciences [15], and natural computing [22] in combination with cultural practices, such as Speculative Design [74] and Bio Design [66]. Such hybrids are creating the context for new forms of human development, such as Dune by Magnus Larssen, is a biological and architectural hybrid that proposes a giant bacterial biofilm printer could transform sand into sandstone in the Sahara and, therefore, combat desertification [66] (pp. 63–65). By engaging material transformation as a property of lively matter as a technical strategy that enhances the biotic activity on which our survival depends, such emerging ideas are developing new design and engineering practices that render industrial systems incomplete, rather than obsolete.

### 3.12. Ethics and Values

The technical systems of the natural world are not yet culturally “real”, in that they have not yet been productized and are therefore not generally accessible. However, it is nonetheless vital to question the value of raising questions about protocell applications as they could potentially offer paradigm-changing tactics in the design and construction of our living spaces.

A technical platform that not only works with life-like properties as a material substrate but also embodies the processes of life, opens up a broad range of key cultural issues, such as the nature of life, our relationship with the environment, the role of design, the ethics of working with life-like technologies and creates new economic opportunities. While it is not the intention of this essay to address these issues in detail, it is certainly worth outlining critical texts that relate to the complex philosophical, cultural and ethical challenges that are invoked by the convergence of natural and artificial systems, which raise many questions about how the human and nonhuman world relate to each other and confront established paradigms of territory and power. These are explored in depth in a range of texts, such as Bruno Latour’s notion of Actor Network Theory that takes a systems view of human and nonhuman relationships [75], Donna Haraway’s companion species manifesto, which examines the ethics of human and nonhuman relations [76], Myra Hird’s notion of microontologies that observes the bacterial world as being a precursor of our own societies [77], Jane Bennett’s proposal for political ecologies that restructure relationships between human and nonhuman communities [51], and Michael Mautner’s directed panspermia manifesto, which creates allegiances between humans and all other life forms [78].

## 4. Conclusions

As an example of natural computing, protocell technology enables life-like strategies to be directly employed in design and engineering solutions. Indeed, it offers something potentially revolutionary to our platforms for human development by liberating the radical creativity of the material realm in ways that transform our surroundings into places that are livelier than they were before we encountered them. However, for protocell technology to be effectively adopted in design practice requires a re-structuring of our urban infrastructures to prioritize the flow of elemental systems that carry energy and matter through our living spaces. Such structures would, in themselves generate new opportunities in working with life-like systems that have the potential to give rise to spontaneous forms of order that ultimately, even be recognized as new kinds of “life”. Engaging a spectrum of life-like and fully alive systems as a technical system are fundamental for an ecological age of design in which the liveliness of the material world is not only appreciated and conserved but may also be augmented—both in terrestrial and non terrestrial environments. Indeed, at this critical juncture in our existence, protocell technology may help us develop an immediate (re)imagining of our world, notions of life, community and what it means to be human in an emerging ecological age, so that we may collectively establish a new platform to underpin human development and increase the vitality of the spaces around us.
